# First Exploration of the Altered Microbial Gut–Lung Axis in the Pathogenesis of Human Refractory Chronic Cough

**DOI:** 10.1007/s00408-024-00681-7

**Published:** 2024-03-25

**Authors:** Simone Baldi, Alessio Fabbrizzi, Leandro Di Gloria, Marco Pallecchi, Giulia Nannini, Mario D’Ambrosio, Cristina Luceri, Gianluca Bartolucci, Matteo Ramazzotti, Giovanni Fontana, Claudia Mannini, Federico Lavorini, Amedeo Amedei

**Affiliations:** 1 Department of Experimental and Clinical Medicine, University of Florence, Largo Brambilla 3, 50134 Florence, Italy; 2 Department of Respiratory Physiopathology, Palagi Hospital, 50122 Florence, Italy; 3 Department of Biomedical, Experimental and Clinical Sciences “Mario Serio”, University of Florence, 50134 Florence, Italy; 4 Department of Neuroscience, Psychology, Drug Research and Child Health NEUROFARBA, University of Florence, 50139 Florence, Italy; 5 Enteric Neuroscience Program, Department of Medicine, Section of Gastroenterology and Hepatology, Mayo Clinic, Rochester, MN 55905 USA; 6 SOD of Interdisciplinary Internal Medicine, Azienda Ospedaliera Universitaria Careggi (AOUC), 50134 Florence, Italy

**Keywords:** Refractory chronic cough, Lung microbiota, Gut microbiota, Gut–lungs axis, Short-chain fatty acids, Free fatty acids

## Abstract

**Purpose:**

Cough represents a natural mechanism that plays an important defensive role in the respiratory tract, but in some conditions, it may become persistent, nonproductive, and harmful. In general, refractory chronic cough (RCC) occurs in about 20% of individuals; hence, we aimed to assess the presence of altered gut–lung communication in RCC patients through a compositional and functional characterization of both gut (GM) and oral microbiota (OM).

**Methods:**

16S rRNA sequencing was used to characterize both GM and OM composition of RCC patients and healthy controls (HC). PICRUST2 assessed functional changes in microbial communities while gas chromatography was used to evaluate fecal short-chain fatty acid levels and serum-free fatty acid (FFA) abundances.

**Results:**

In comparison with HC, RCC patients reported increased saliva alpha-diversity and statistically significant beta-diversity in both GM and OM. Also, a, respectively, significant increased or reduced Firmicutes/Bacteroidota ratio in stool and saliva samples of RCC patients has been shown, in addition to a modification of the abundances of several taxa in both GM and OM. Moreover, a potential fecal over-expression of lipopolysaccharide biosynthesis and lipoic acid metabolism pathways and several differences in serum FFA levels have been reported in RCC patients than in HC.

**Conclusion:**

Since differences in both GM and OM of RCC patients have been documented, these findings could provide new information about RCC pathogenesis and also pave the way for the development of novel nutritional or pharmacological interventions for the management of RCC through the restoration of eubiotic gut–lung communication.

**Supplementary Information:**

The online version contains supplementary material available at 10.1007/s00408-024-00681-7.

## Introduction

Chronic cough, that is a cough lasting longer than eight weeks, is among the most common respiratory symptoms for which patients seek medical advice [1]. It has long been recognized that virtually all diseases of the respiratory tract, as well as some non-respiratory disorders, are accompanied by chronic cough and that a relevant percentage of patients may suffer from a long-lasting cough for which no respiratory or extra-respiratory cause can be identified. Although the majority of patients can benefit from treatments of the underlying cause(s), it is increasingly recognized that cough does not improve with such treatments in a large percentage of patients; these patients are commonly classified as having refractory chronic cough (RCC). In a minority of patients, the cough remains unexplained even after accurate investigations, and these patients are considered to be suffering from unexplained chronic cough (UCC) [2]. Subjects with RCC and UCC are currently believed to be affected by a condition known as the “cough hypersensitivity syndrome,” in which the physiological cough reflex becomes hypersensitized to stimuli that are inoffensive to the normal population [3].

The mechanisms underlying hypersensitization are still poorly defined and may involve both peripheral and central (medullary) neural structures that are crucial for producing the cough motor pattern, potentially representing a strategic site of action for antitussive drugs. Patients with RCC or UCC are predominantly women (80%), mostly postmenopausal, and are eight times more likely to have an organ-specific autoimmune disease, especially hypothyroidism [4, 5]. In general, due to various environmental factors, RCC/UCC prevalence is significantly higher in Europe and America than in Asia and Africa [6]; in addition, certain comorbidities (e.g., obesity, rhinitis) and smoking or alcohol abuse may contribute to increased regional RCC/UCC variability [7].

Recent evidence has suggested that the lung microbiome (LM) plays a critical role in the development and progression of various respiratory diseases [8]; hence, its involvement in RCC/UCC pathogenesis could be hypothesized [9]. Until a few years ago, lungs were considered a sterile district; however, their microbial population has been recently documented to be equivalent to that of the duodenum one [10]. Usually, although healthy people display a different microbial composition between the upper and lower respiratory tract, the most LM predominant phyla are Bacteroidetes, Firmicutes, Proteobacteria, and Actinobacteria while *Prevotella*, *Streptococcus*, *Veillonella*, *Neisseria*, *Haemophilus*, and *Fusobacterium* are the most abundant genera [11, 12].

Moreover, emerging experimental and epidemiological evidence has highlighted the existence of a crucial cross-talk between the gut microbiota (GM) and the lungs that is currently known as the ‘gut–lung axis’ [13]. Perturbations of the GM composition and/or function, referred to as dysbiosis, are linked with altered immunity and homeostasis in the airways. For instance, an altered gut–lung axis has been associated with increased susceptibility to airway diseases and infections; as an explicative example, we can cite that the patients affected by inflammatory bowel disease usually show a higher prevalence of pulmonary diseases [14, 15]. In addition, various microbial-derived metabolites, especially short-chain fatty acids (SCFA), have been documented to play a pivotal role in regulating the immune system in both the intestine and the airways [16]. Specifically, SCFAs exert anti-inflammatory and immune-modulatory effects by i) acting as potent ligands of G protein-coupled receptors and ii) inhibiting histone deacetylase (HDAC) activity in various cell types and tissues [17, 18]. Furthermore, the SCFAs can also pass through the intestinal epithelium entering the bloodstream and reaching the lungs where they promote an extrathymic peripheral Treg (T regulatory cells) pool, linked to dampening allergic airway diseases through HDAC inhibition [19].

In this scenario, we have performed a compositional and functional characterization of intestinal and saliva microbiota of RCC patients in order to evaluate the presence of an intestinal or oral dysbiosis that might help to understand the pathogenesis of RCC/UCC paving the way for novel topic investigations.

## Materials and Methods

### Patients

We enrolled 10 adult non-smoking outpatients with an established diagnosis of RCC and 10 gender-and age-matched healthy volunteers as controls (HC). For each patient, data collection and routine clinical assessments were performed in accordance with international guidelines [20]. None of the patients reported a history of recent (< 4 weeks) respiratory infection nor had evidence of active lesions documented by a recent (within 2 months) chest X-ray. Patients rated the magnitude of their cough disturbance using a 0–9 modified Borg Scale where 0 indicated “Not bothered at all” and 9 indicated “Worst disturbance I can possibly imagine”; the values obtained with this method were termed as “cough score” [21].

Subjects were also excluded if they were taking medications or probiotics and similar (prebiotics or symbiotics) or traveled to an exotic region in the last three months, were pregnant or lactating or had a serious illness or unstable condition.

This study was approved by the Ethics Committee of the Careggi University Hospital (OSS-1431) and followed the principles of the Declaration of Helsinki. The patients gave their written informed consent.

### Fecal and Salivary Microbiota Characterization

The genomic DNA was extracted from frozen (−80 °C) stool and saliva samples using the DNeasy PowerSoil Pro Kit (Qiagen, Hilden, Germany), following the manufacturer’s instructions. For saliva samples, a pre-processing step involved centrifugation in a 1.5-mL microcentrifuge tube at 10.000 rpm for 10 min, discarding the supernatants and collecting the pellets. Briefly, 0.25 g of stool sample or the salivary pellet was added to a bead-beating tube and homogenized with TissueLyser LT (Qiagen, Hilden, Germany) for 5 min at 50 Hz. Afterward, DNA was captured on a silica membrane in a spin column format, washed, and eluted. The quality and quantity of extracted DNA were assessed with both NanoDrop ND-1000 (Thermo Fisher Scientific, Waltham, USA) and Qubit Fluorometer (Thermo Fisher Scientific, Waltham, USA) and then it was stored at −20 °C. Subsequently, total DNA samples were sent to IGA Technology Services (Udine, Italy) where amplicons of the variable V3–V4 region of the bacterial 16S rRNA gene (341F:CCTACGGGNGGCWGCAG; 805R: GACTACNVGGGTWTCTAATCC) were sequenced in paired-end (2 × 300 cycles) on the Illumina MiSeq platform, according to the Illumina 16S Metagenomic Sequencing Library Preparation protocol.

Demultiplexed sequence reads were processed using QIIME2 2022.8 [22]. The sequencing primers and the reads without primers were removed using the Cutadapt tool v3.4 [23] while DADA2 [24] was used to perform paired-end reads filtering, merging, and chimeras removal steps after trimming low-quality nucleotides from both forward and reverse reads (-p-trunc-len-f 250 and -p-trunc-len-r 204).

Hence, ASVs (amplicon sequence variants) were generated and the taxonomic assignments were performed through the Scikit-learn multinomial naive Bayes classifier re-trained on the SILVA database (release 138) V3–V4 hypervariable region. Each cross-amplified host DNA was identified by aligning the ASVs to GRCh38 (human reference genome) using Bowtie2 v.2.2.5. ASVs associated with genera with maximum relative abundance across the samples under the cut-off of 0.005% have been discarded to minimize sequencing contaminants and improve statistical inferences [25, 26].

Further details about the FASTQ processing are available at github.com/LeandroD94/Papers/tree/main/2023_Idiopathic_Chronic_Cough_batteriota.

### Fecal SCFA Evaluation by GC–MS Analysis

The qualitative and quantitative evaluation of fecal SCFA was performed by an Agilent gas chromatography-mass spectrometry (GC–MS) system composed of 5971 single quadrupole mass spectrometer, 5890 gas chromatograph, and 7673 autosampler, through our previously described method [27].

Briefly, just before the analysis, stool samples were thawed and combined a with 0.25 mM sodium bicarbonate solution (1:1 w/v) in a 1.5-mL centrifuge tube. The resulting suspensions were sonicated for 5 min and centrifuged at 5.000 rpm for 10 min, and then the supernatants were collected. The SCFAs were finally extracted as follows: an aliquot of 100 µL of sample solution (corresponding to 0.1 mg of stool sample) was added to 50 µL of internal standards mixture, 1 mL of tert-butyl methyl ether, and 50 µL of HCl 6 M + 0.5 M NaCl solution in a 1.5-mL centrifuge tube. Subsequently, each tube was shaken in a vortex apparatus for 2 min and centrifuged at 10.000 rpm for 5 min, and lastly, the solvent layer was transferred to an autosampler vial and processed three times.

### Serum-Free Fatty Acids Quantification by GC–MS Analysis

Free fatty acids (FFAs), namely circulating SCFA, medium-chain fatty acids (MCFAs), and long-chain fatty acids (LCFAs), were analyzed using our previously described GC–MS protocol [28, 29]. Briefly, just before the analysis, each sample was thawed and the FFAs were extracted as follows: an aliquot of 200 μL of serum sample was added to 10 μL of ISTD mixture, 100 μL of tert–butyl methyl ether, and 20 μL of 6 M HCl + 0.5 M NaCl solution in a 0.5 mL centrifuge tube. Afterward, each tube was stirred in a vortex for 2 min and centrifuged at 10,000 rpm for 5 min, and finally, the solvent layer was transferred to a vial with a microvolume insert and analyzed.

### Statistical Analysis

The statistical analyses on bacterial communities were performed in R 4.2.1 with the help of the packages phyloseq 1.44.0 [30], vegan 2.6–4, DESeq2 1.40.1 [31] and other packages satisfying their dependencies. The packages ggplot2 3.4.2, ggh4x 0.2.2 and ggpubr 0.40 were used to plot data and results. A saturation analysis on ASV was performed on every sample using the function rarecurve (step 100 reads), further processed to highlight saturated samples (arbitrarily defined as saturated samples with a final slope in the rarefaction curve with an increment in ASV number per reads < 1e-5). The observed richness and Shannon indices were used to estimate the bacterial alpha-diversity in each sample using the function estimate_richness from phyloseq.

The Pielou’s evenness index was calculated using the formula E = S/log(R), where S is the Shannon diversity index and R is the observed ASV richness in the sample. Differences in alpha-diversity indices and the Firmicutes/Bacteroidetes ratio were inspected using the Mann–Whitney test. PCoAs were performed using the Hellinger distance on Hellinger transformed genera abundances. PERMANOVA and Betadisper were used to test the statistical significance of the beta-diversity distances and dispersions. At different taxonomic ranks, the differential analysis of the abundances was computed with DESeq2 on raw count data. Furthermore, differentially abundant taxa with a DESeq2 baseMean value < 50 have been discarded from the displayed results, irrespective of their statistical significance to limit noisy results. Moreover, potentially expressed KEGG pathways in each group were predicted through PICRUST2 v2.5 with the SEPP algorithm and then significant differences were explored using LEFSE 1.1.2 (LDA Effect Size) analysis [32]. Only the results with a log10 LDA score over 3 were considered. Finally, the monotonic relationships between DA genera relative abundances and serum FFA levels among RCC patients were explored using Spearman correlation. Every *p*-value related to multiple tests has been adjusted according to the Benjamini–Hochberg method.

Furthermore, the software GraphPad Prism was used for the statistical analysis of fecal SCFA and MCFA levels and serum FFA abundances between RCC patients and HC; differences were assessed using the Mann–Whitney test and p-values less than 0,05 were considered statically significant.

Further details about the data analysis are available at github.com/LeandroD94/Papers/tree/main/2023_Idiopathic_Chronic_Cough_batteriota.

## Results

### Enrolled Patients

Demographical and clinical features of patients and HC are reported in Table 1.

**Table 1:** Demographical and clinicopathological features of enrolled RCC patients

Sample ID	Gender	Age	BMI	Smoke	Cough score
RCC 1	F	56	22.4	No	9
RCC 2	F	63	27.0	Ex	9
RCC 3	F	68	22.8	No	7
RCC 4	F	76	19.4	No	5
RCC 5	F	52	22.7	Ex	6
RCC 6	F	56	26.8	Ex	5
RCC 7	F	81	36.0	No	7
RCC 8	F	66	22,2	Ex	6
RCC 9	F	70	24,5	Ex	7
RCC 10	F	67	23.8	No	7

RCC: refractory chronic cough, BMI: body mass index

All patients were female, with a mean age of 65,5 (range 52–81) and a BMI of 23,35 ± 1,62 kg/m^2^. The mean cough score was 6,8 (range 5–9). At enrollment, patients were treated with cough suppressants such as Gabapentin, central (Codeine), or peripheral antitussive drugs (Levodropropizine). No gut diseases or intolerances were diagnosed in any enrolled patients.

### Gut and Oral Microbiota Composition

**Fig. 1:**
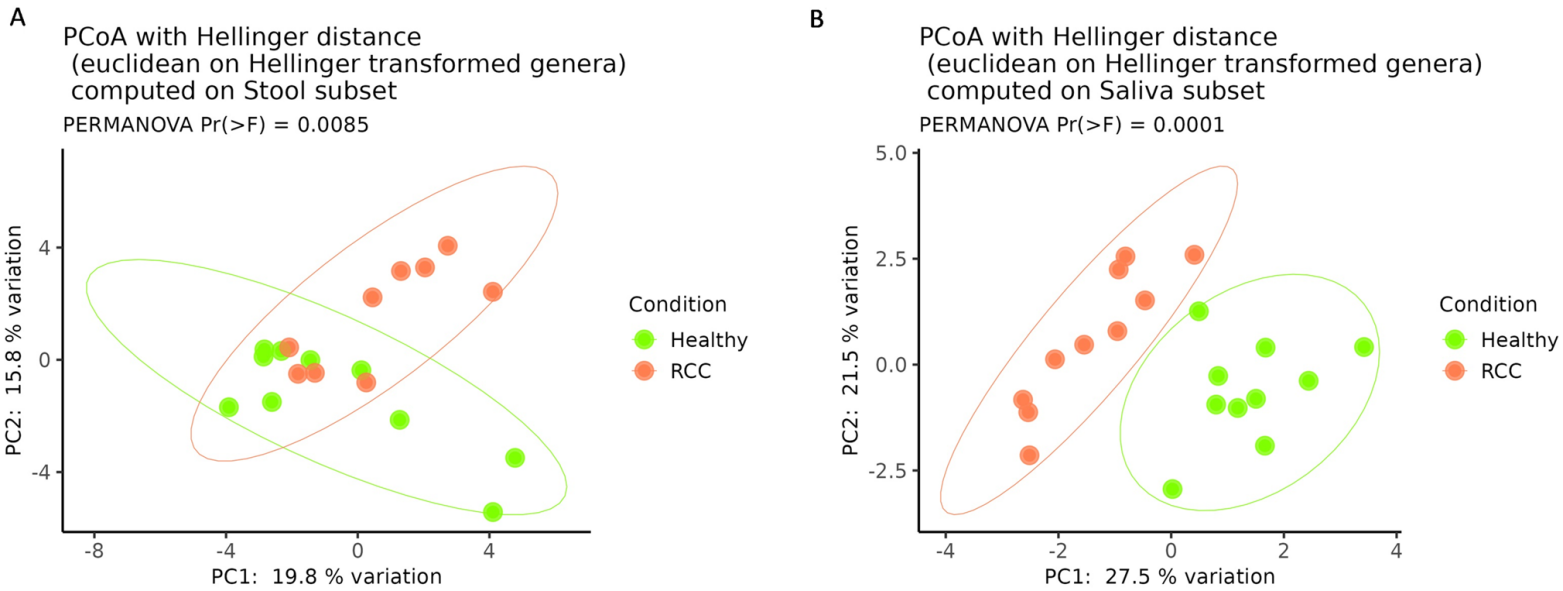
Principal coordinate analysis (PCoA) conducted with the Hellinger distance on transformed genera abundances of stool (**A**) and saliva (**B**) samples among HC and RCC patients. RCC: refractory chronic cough, HC: healthy controls

Rarefaction curves for observed ASVs indicated that both stool and saliva specimens were sufficiently sampled (Figure S1). In particular, the principal coordinate analysis computed using the Hellinger distance on transformed genera abundances highlighted a separation among stool (PERMANOVA, p < 0,0085) (Fig. 1A) and saliva samples (PERMANOVA, p < 0.0001) (Fig. 1B) of both HC and RCC patients.

Of note, statistically significant beta-diversities between stool and saliva samples of HC and RCC patients were also found at all other taxonomic ranks (Table S1).

**Fig. 2:**
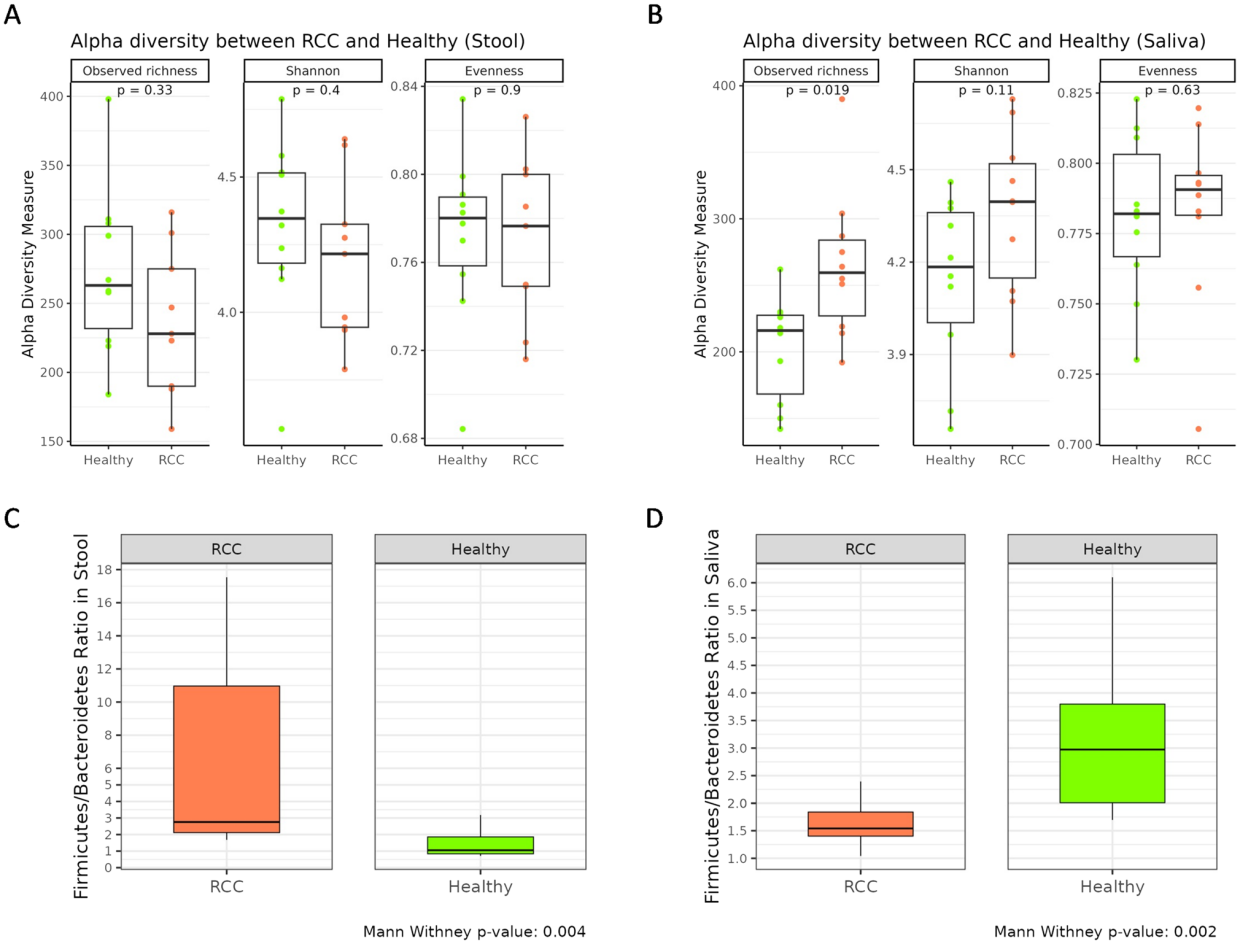
Box plots showing alpha-diversity indices (Observed ASV, Shannon index, Pielou’s evenness) of stool (**A**) and saliva (**B**) samples and the fecal (**C**) and saliva (**D**) F/B ratio among HC and RCC patients. RCC: refractory chronic cough, HC: healthy controls

Although no significant differences in the alpha-diversity indices were reported between stool samples of HC and RCC patients (Fig. 2A), an increased saliva alpha-diversity (observed ASV richness, *p* = 0.019) was documented in patients in comparison to HC (Fig. 2B), Additionally, a significant (*p* = 0.004) increase in the fecal Firmicutes/Bacteroidota (F/B) ratio in the patients compared to HC was observed (Fig. 2C); conversely, the RCC patients showed a significantly (*p* = 0.002) lower saliva F/B ratio than HC (Fig. 2D).

Regarding the taxonomic analysis, the stacked bar plots depicted different relative abundances of the top five phyla and the top eight genera in either stool (Figures S2A and S2B) and saliva (Figures S2C and S2D) samples collected from RCC patients and HC.

In more detail, the top five represented phyla in stool samples were Actinobateriota, Bacteroidota, Firmicutes, Proteobacteria, and Verrucomicrobiota while saliva samples showed high abundances of Actinobateriota, Bacteroidota, Firmicutes, Fusobacteriota, and Proteobacteria. Besides, the top eight represented genera in stool samples were *Alistipes*, *Bacteroides*, *Bifidobacterium*, *Blautia*, *Faecalibacterium*, *Prevotella*, *Ruminococcus,* and *Subdoligranulum* whereas saliva samples showed high abundances of *Fusobacterium*, *Haemophilus*, *Neisseria*, *Porphyromonas*, *Prevotella*, *Rothia*, *Streptococcus,* and *Veillonella*.

**Fig. 3:**
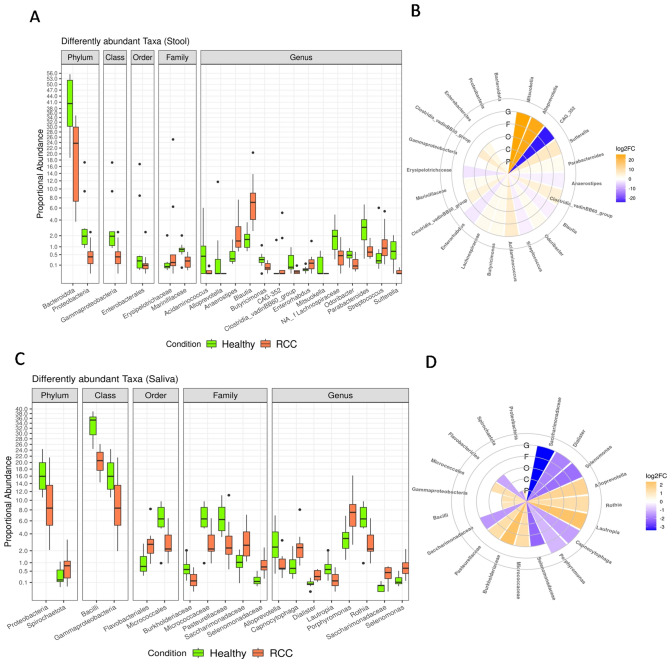
Boxplot (**A**) and circoplot (**B**), respectively, showing the results of differential abundances analysis and log2fold change between taxa of the stool (Panels A, B) and saliva (Panels C, D) samples from HC and RCC patients. Letters indicate the taxonomic depth, in detail, G = genus, F = family, O = order, C = class, P = phylum. All results have an FDR < 0.05

Notably, several taxa resulted differentially abundant in stool (Fig. 3A, B and Table S2) and saliva (Fig. 3B, C and Table S3) samples of HC and RCC patients.

More precisely, compared to HC, RCC patients reported higher fecal abundances of Erysipelotrichaceae, *Anaerostipes spp.*, *Blautia spp.*, *CAG-352 spp.*, *Enterorhabdus spp.,* and *Streptococcus spp.* as well as reduced abundances of Bacteroidota, Proteobacteria, Gammaproteobacteria, Enterobacterales, Marinifilaceae, *Acidaminococcus spp.,* *Alloprevotella spp.*, *Butyricimonas spp.*, *Clostridia_vadinBB60_group spp.*, *Mitsuokella spp.*, unidentified genus of Lachnospiraceae family, *Odoribacter spp.*, *Parabacteroides spp., and Sutterella spp.*

On the other hand, RCC patients showed higher saliva levels of Spirochaetota, Flavobacteriales, Saccharimonadaceae, Selenomonadaceae, *Capnocytophaga spp.*, *Dialister spp.*, *Porphyromonas spp.*, *Saccharimonadaceae spp.,* and *Selenomonas spp.* but lower levels of Proteobacteria, Bacilli, Gammaproteobacteria, Micrococcales, Burkholderiaceae, Micrococcaceae, Pasteurellaceae, *Alloprevotella spp.*, *Lautropia spp.,* and *Rothia spp.* than HC.

### Functional Analysis of the Fecal and Oral Microbiota

**Fig. 4:**
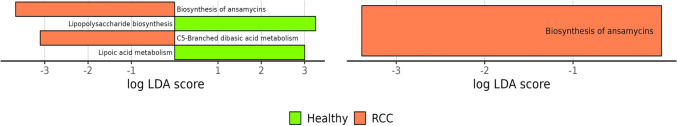
Significant enriched KEGG pathways with LDA score > 3.0 in stool (**A**) and saliva (**B**) samples of HC and RCC patients. Pathways more abundant in HC are indicated with a positive LDA score (green) while pathways more abundant in RCC patients are indicated with a negative LDA score (red). RCC: refractory chronic cough, HC: healthy controls, LDA score: Linear discriminant analysis effect size

The PICRUSt2 (phylogenetic investigation of communities by reconstruction of unobserved states) [33] predictive metabolism approach was used on the 16S rRNA gene sequencing data to assess a functional GM analysis of RCC patients and HC. Using the KEGG metabolic pathway database, RCC patients exhibited a potential upregulated fecal profile in lipopolysaccharide biosynthesis (*p* = 0.002) and lipoic acid metabolism (*p* = 0.017) compared to HC. On the contrary, for HC, in comparison with RCC patients, potential higher expressed fecal pathways in the biosynthesis of ansamycins (*p* = 0.001) and C5-Branched dibasic acid metabolism (*p* = 3,2e−4) were predicted (Fig. 4A).

Finally, regarding the saliva samples, the biosynthesis of ansamycins (*p* = 0.001) pathway resulted potentially higher expressed in RCC patients than in HC (Fig. 4B).

### Analysis of Fecal SCFA and Serum FFA

Microbial-derived SCFA abundances in fecal samples of HC and RCC patients were assessed with a GC–MS protocol. Since these analyses could be in part influenced by the total amount of each metabolite, we performed the comparisons on the SCFA percentage compositions (Table S4); however, no statistically significant differences were found between RCC patients and HC.

**Fig. 5:**
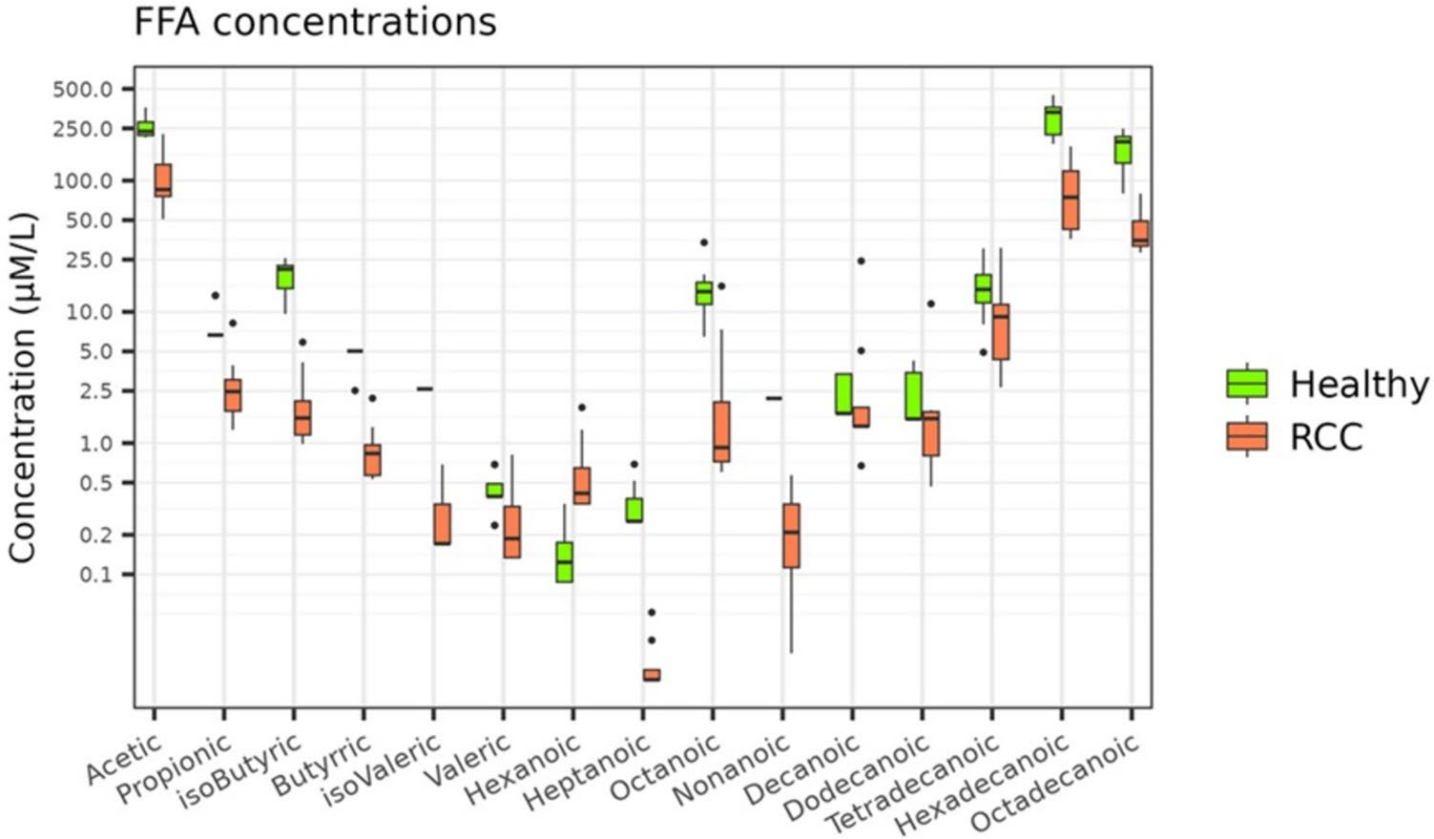
Boxplots representing each FFA percentage in RCC patients and HC

On the other hand, several differences in serum FFAs levels were observed. Specifically, RCC patients, compared to HC, exhibited significantly increased levels of hexanoic acid and significantly reduced levels of acetic, propionic, butyric, isobutyric, isovaleric, heptanoic, octanoic, nonanoic, hexadecanoic, and octadecanoic acids. Figure 5 displays each FFA level for RCC patients and HC while the statistical results of the comparisons conducted for FFA abundances are shown in Table 2.

**Table 2:** Serum FFAs abundances of HC and RCC patients

FFAs (μmol/L)	HC	RCC	*p*-values
SCFAs (μmol/L)			
Acetic acid	255.03 ± 47.41	107.88 ± 56.81	0.0004
Propionic acid	7.99 ± 2.81	3.04 ± 2.25	0,0034
Isobutyric acid	18.59 ± 5.66	0.96 ± 0.56	0.0004
Butyric acid	4.77 ± 0.78	2.25 ± 1.77	0.0004
Isovaleric acid	2.58 ± 0.01	0.28 ± 0.18	0.0004
Valeric acid	0.43 ± 0.15	0.31 ± 0,27	0,1154
MCFAs (μmol/L)			
Hexanoic	0.14 ± 0.08	0.69 ± 0.57	0.0006
Heptanoic	0.34 ± 0.15	0.02 ± 0.01	0.0002
Octanoic	15.48 ± 7.41	3.53 ± 5.42	0.0021
Nonanoic	2.19 ± 0.00	0.24 ± 0.18	0.0004
Decanoic	2.35 ± 0.88	4.61 ± 8.11	0.0728
Dodecanoic	2.39 ± 1.16	2.51 ± 3.67	0.2980
LCFAs (μmol/L)			
Tetradecanoic	15.65 ± 7.38	10.46 ± 8.91	0.0676
Hexadecanoic	306.36 ± 85.34	90.23 ± 56.33	0.0001
Octadecanoic	181.85 ± 58.62	44.22 ± 19.30	0.0001

*p*-values were assessed the with Mann–Whitney test

FFAs: free fatty acids, SCFAs: short-chain fatty acids, MCFAs: medium-chain fatty acids, LCFAs: long-chain fatty acids HC: healthy control, RCC: refractory chronic cough

Finally, Spearman correlations were performed between differentially abundant fecal and oral taxa and significant serum FFA abundances to evaluate potential associations between taxonomy and function, providing insights into their role in RCC pathogenesis.

**Fig. 6:**
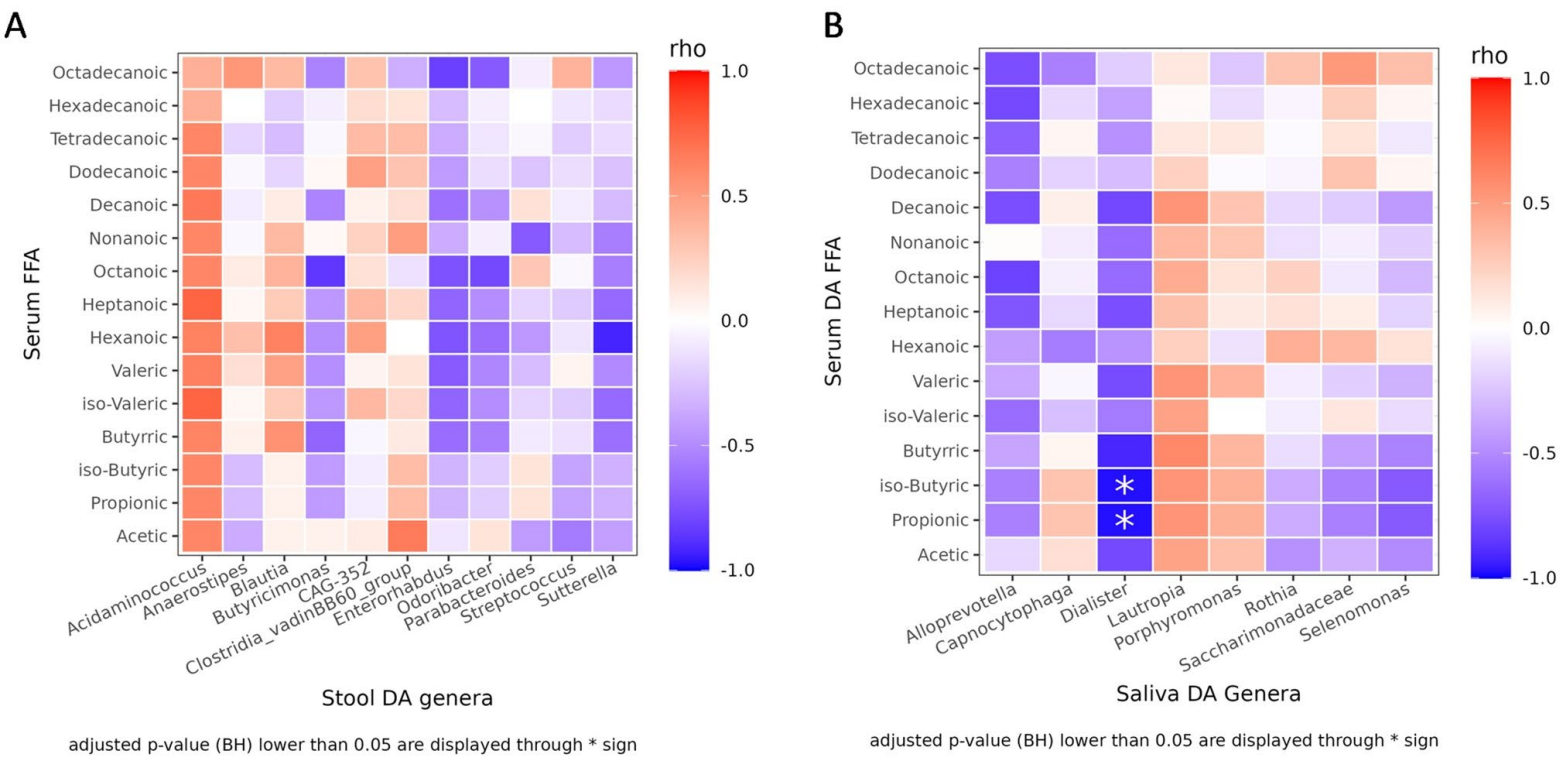
Heatmaps of Spearman correlations between serum FFAs abundances and differentially abundant fecal (**A**) and oral (**B**) taxa among RCC patients and HC. Red shades indicate positive correlations, whereas blue shades indicate negative correlations; the intensity of colors represents the degree of association. *p*-values adjusted according to the Benjamini–Hochberg method less than 0.05 were considered statistically significant

Although no correlation has been reported between gut bacteria and serum FFAs (Fig. 6A), a strong negative correlation was observed between saliva *Dialister spp.* and both propionic (padj = 0.003) and isobutyric (padj = 0.003) acids in RCC patients (Fig. 6B).

## Discussion

The respiratory tract, previously considered sterile, is one of the latest body sites being explored for the characterization of human-associated microbial communities. LM is a dynamic ecosystem whose composition in healthy lungs is likely to reflect microbial migration, elimination, and reproduction.

In detail, although certain bacteria are more abundantly represented in the airway microbiota than in the corresponding OM, primarily due to a selective advantage in replicating in the lung microenvironment compared to the oral one, a close resemblance of the LM to the OM has been documented [34]. The microbiome’s continuity in the lower respiratory tract is likely defined by the entry of bacteria into the lungs via regular OM microaspirations; conversely, LM members can propagate to the OM through coughing [35].

Moreover, it is now widely accepted that LM, intimately related to the GM, undergoes alterations in various respiratory disorders such as obstructive airway diseases [36], interstitial lung diseases [37], infections [38], and lung cancer [39]. Hence, we hypothesized a potential involvement of LM also in the pathogenesis of RCC/UCC.

Consequently, through a compositional and functional characterization of both intestinal and saliva microbiota of patients with RCC, we found, for the first time, that, compared with HC, patients presented a significant increase in microbiota alpha-diversity in saliva but not in stool samples. Significant differences in microbiota beta diversities were also observed between chronic coughers and HC in both intestinal and oral microbiota. Taken together, these findings support previous reports suggesting that gut and lungs are linked organs and changes in the GM community can influence the LM and vice versa [13, 40].

For instance, modification in newborns’ diet influences the composition of their LM while fecal transplantation in rats induces changes in their LM [41]. Moreover, the LM becomes enriched with gut bacteria after sepsis [42] and LPS instillation in the lungs of mice is associated with GM disturbances [43].

Consisted with our findings, a significant increase in oral alpha-diversity has been reported in patients with asthma [44] or COPD [45] compared with HC. However, no significant differences have been reported in fecal alpha-diversity indices among COPD patients and healthy subjects [46]. Moreover, a significant parting of the intestinal and saliva microbiota among RCC patients and HC has been documented.

Finally, in comparison with HC, RCC patients reported a significant increase in the fecal F/B ratio and a significant decrease in the saliva F/B ratio. In particular, an increased fecal F/B ratio has been associated with elevated lung IL-17 and IL-22 responses and enhanced airway hyperresponsiveness [47]. In general, these microbial compositional alterations in both oral and intestinal microbiota of RCC patients reflect the presence of a remarkable dysbiosis condition.

The analysis performed at all taxonomic ranks as in stool as in saliva samples also revealed significant differences in several taxa between RCC patients and HC. In particular, RCC patients reported higher fecal abundances of Erysipelotrichaceae family and *Anaerostipes*, *Blautia.*, *CAG-352*, *Enterorhabdus,* and *Streptococcus* genera*.* In line with our findings, Erysipelotrichaceae members increased in COPD patients [45] but, in contrast to our results, *Blautia*, *Anaerostipes,* and *Streptococcus* genera were reduced in the GM of patients with cystic fibrosis or COPD [48, 49]. Moreover, we documented reduced abundances of bacteria belonging to Bacteroidota and Proteobacteria phyla, Gammaproteobacteria class, Enterobacterales order, Marinifilaceae family, and *Acidaminococcus*, *Alloprevotella*, *Butyricimonas*, *Clostridia_vadinBB60_group*, *Mitsuokella*, *Odoribacter*, *Parabacteroides*, and *Sutterella* genera in RCC patients compared to HC.

Regarding differences at the phylum level*, *Bacteroidota members are known to be overrepresented in healthy people [50] while Proteobacteria have been reported as relevant producers of lipopolysaccharide (LPS), which is in turn implicated in COPD development [51]. Notably, reduced levels of Enterobacteriaceae and Acidaminococcaceae have been reported in asthmatic patients [52, 53] while Lai et al. highlighted a significant negative association between *Parabacteroides goldsteinii* and COPD severity [54]. Additionally, Chiu et al., documented a lower abundance of *Alloprevotella spp.* in patients with rapid lung function decline [46] while a decreased abundance of *Odoribacter spp.* has been linked to different microbiota-associated diseases, such as inflammatory bowel disease and cystic fibrosis [55]. Finally, lower levels of *Butyricimonas spp.* have been associated with a detrimental decrease of butyric-acid production, a renowned SCFA with potent anti-inflammatory properties [56].

Concerning the saliva samples, RCC showed higher saliva levels of members of Spirochaetota phylum, Flavobacteriales order, Saccharimonadaceae and Selenomonadaceae families, and *Capnocytophaga*, *Dialister*, *Porphyromonas*, *Saccharimonadaceae,* and *Selenomonas* genera than HC*.*

Notably, Spirochaetota and *Porphyromonas* species have been widely associated with the pathogenesis of the periodontal disease [57, 58], a condition that may worsen COPD outcomes and play a causal role in the occurrence of pneumonia and bronchitis.

In contrast, Flavobacteriales, *Capnocytophaga, Dialister,* and *Selenomonas* species were significantly increased in the LM of COPD patients [45, 59].

In addition, our results showed reduced levels of bacteria belonging to Proteobacteria and Bacilli phyla, Gammaproteobacteria class, Micrococcales order, Burkholderiaceae, Micrococcaceae and Pasteurellaceae families, and *Alloprevotella, Lautropia,* and *Rothia* genera in RCC compared to HC.

In line with these findings, the relative abundance of Gammaproteobacteria, Bacilli, and Micrococcaceae members were decreased in asthmatic patients [60, 61] while lower levels of *Rothia mucilaginosa*, a common bacteria having inhibitory effects on pathogen- or LPS-induced pro-inflammatory responses, have been reported in patients with chronic lung disease [62].

Furthermore, to better characterize the consequences of these changes in both intestinal and oral microbiota, we performed a predictive functional analysis using the PICRUSt2 software. In detail, compared to HC, RCC patients showed a potential upregulation in the fecal pathways of lipopolysaccharide biosynthesis and lipoic acid metabolism but a lower biosynthesis of ansamycins, which conversely resulted potentially upregulated in saliva samples of RCC patients.

LPS is among the most potent microbial inducers of inflammation and is implicated in the deleterious effects of pulmonary infections. Animal models have reported that ML-7, a potent myosin light-chain kinase (MLCK) inhibitor, impedes neutrophilic inflammation caused by LPS in various respiratory diseases [63]. Interestingly, RCC patients exhibited a high metabolism of lipoic acid but its beneficial role in ameliorating many respiratory diseases (e.g., lung cancer, fibrosis, asthma, and acute lung injury) has been suggested because it shows anti-oxidative and anti-inflammatory properties [64].

On the other hand, ansamycins are secondary metabolites, mainly produced by Actinobacteria, known for their antimicrobial properties and currently used as the first-line treatment of tuberculosis [65]. Our results showed a high representation of Actinobacteria in both fecal and saliva microbiota of RCC patients, yet the potential upregulation of ansamycins biosynthesis was observed only in saliva samples but not in stool.

Finally, a microbial functional evaluation has been assessed through the analysis of fecal SCFA abundances and the evaluation of serum circulating FFAs in RCC patients and HC. About fecal SCFA, no statistically significant differences were found between RCC patients and HC, mainly because no SCFA-producing bacteria resulted differently abundant between groups. However, it’s noteworthy that a significant decrease in the total fecal content of SCFAs has been detected in some lung diseases including COPD and asthma [66, 67].

Anyway, regarding serum FFA abundances, RCC patients showed a significant increase in hexanoic acid, a bacterial metabolite known for its pro–inflammatory role through the activation of p38 MAPK signaling [68]. Moreover, in comparison to healthy subjects, RCC patients reported significantly reduced levels of various SCFA, MCFAs, and LCFAs.

Circulating FFA exerts well-established pleiotropic functions, ranging from maintaining an intestinal–epithelial integrity to dampening inflammation in the gut and respiratory tract [16]. While SCFAs promote the differentiation of immune-suppressive T regs in the gut [69], their detection in the lungs is limited, possibly due to the absence of digestible substrates [16]. However, Trompette et al. documented that, along the gut–lung axis, SCFAs play a protective role against allergic airway diseases and respiratory infection by priming myeloid cells in the bone marrow. These cells subsequently migrate to the lungs, shaping an anti-inflammatory milieu [70].

In RCC patients, we also documented a strong anti-correlation between saliva Dialister *spp.* abundance and serum levels of anti-inflammatory propionic and isobutyric acids. *Dialister* species are known intestinal SCFA producers [71] but an increased abundance of saliva *Dialister spp.* has been associated with oral and lung diseases [72, 73]. Importantly, *Dialister spp.* showed an anti-correlation with serum neutrophil to lymphocyte ratio and platelet lymphocyte ratio; two parameters increased in stable COPD patients [74].

Overall, we speculate that these alterations in intestinal and oral microbiota may play a role in RCC development through a complex cross-talk involving the gut, lungs, and brain. The bidirectional communication between the central and the intestinal nervous system, involving nerves, endocrine pathways, immunity, and microbial interactions, has been widely documented [75], with the bacterial SCFA acting as major metabolites that can affect various central nervous system (CNS) aspects [76].

In detail, SCFAs can directly or indirectly modulate vagal afferent fibers, leading to the activation of efferent fibers that conduct feedback signals from the CNS to the lungs, forming the “brain-lung axis.” This process promotes bronchial smooth muscle contraction, glandular secretion, mucosal swelling, and cough [77, 78]. Furthermore, intestinal and/or pulmonary dysbiosis can be the cause or contributory factor to a systemic and nervous hyperinflammatory state, which, in turn, disrupts both the intestine–brain and brain–lung communication pathways [78, 79].

This study has some limitations, including the restricted number of enrolled patients, and the evaluation of the LM composition only through saliva samples. However, we have documented, for the first time, numerous and consistent differences in the gut and oral microbial communities of RCC patients that could reflect an unbalanced gut–lung communication. Hence, although future studies are needed, these findings introduce new impacting factors in RCC pathogenesis, paving the way for further investigations and the development of novel therapeutic interventions for RCC management based on the modulation of microbial gut–lung communication.

### Supplementary Information

Below is the link to the electronic supplementary material.

Supplementary file1 (DOCX 271 KB)

## Data Availability

The data presented in this study are deposited in the NCBI Gene Expression Omnibus (GEO) repository, accession number GSE240646.
