# Supplementary material for: First Exploration of the Altered Microbial Gut–Lung Axis in the Pathogenesis of Human Refractory Chronic Cough
Source: Lung. 2024 Mar 25;202(2):107–18. doi: 10.1007/s00408-024-00681-7 (PMC11009740; doi:10.1007/s00408-024-00681-7)
Supplement: Supplementary file 1 — Supplementary file1 (DOCX 271 KB) [file 408_2024_681_MOESM1_ESM.docx]

**SUPPLEMENTARY FIGURES**

**Figure S1.** ASVs rarefaction curves of stool and saliva samples of RCC patients and HC.


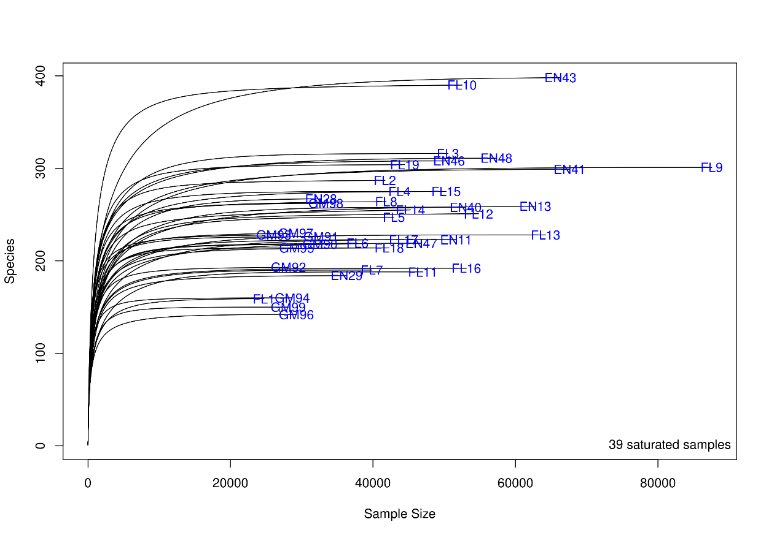


**Figure S2.** Stacked bar plots showing the relative abundances of the five most abundant phyla (A) and the eight most abundant genera (B) in stool samples and the five most abundant phyla (C) and the eight most abundant genera (D) in saliva samples of HC and RCC patients. “Others” includes every taxon below the top five or eight ranks. HC: healthy controls, RCC: refractory chronic cough.

**
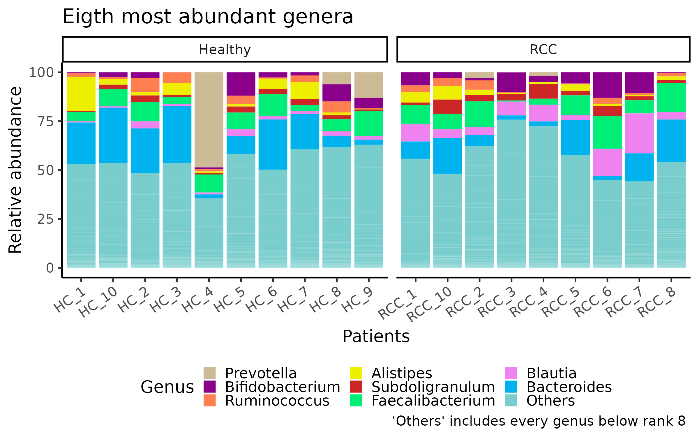

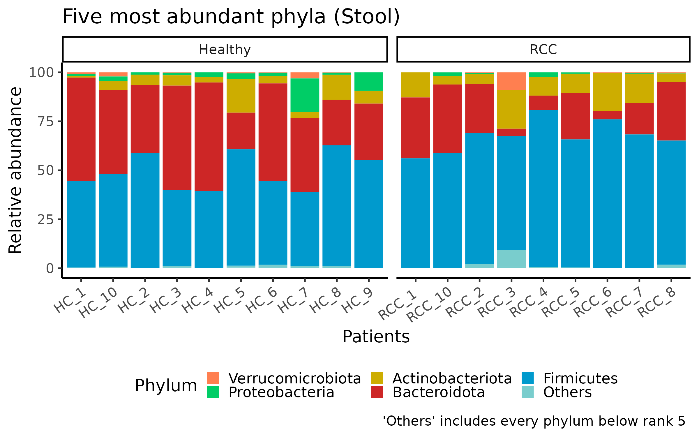
**

A)

B)

C)

D)

**
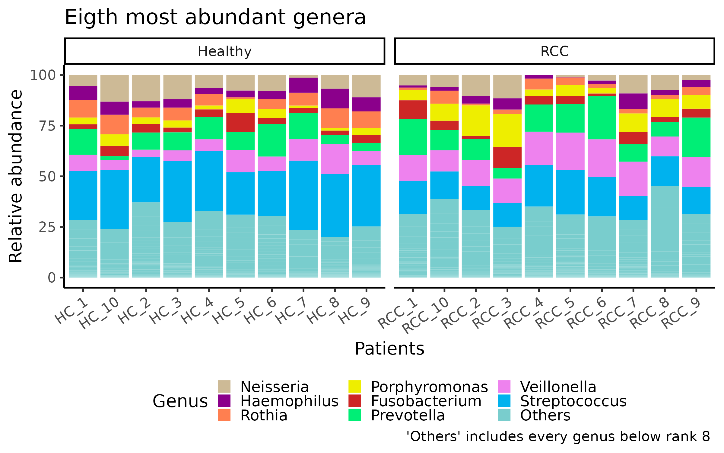

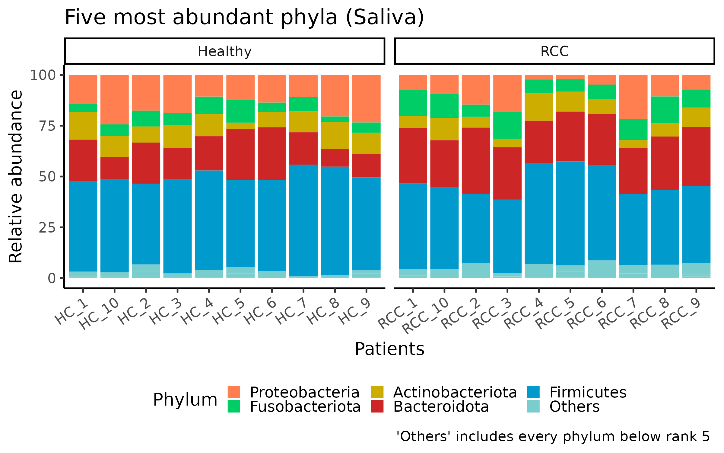
**

**SUPPLEMENTARY TABLES**

**Table S1.** PERMANOVA tests on the Hellinger distance on transformed abundances at all taxonomic ranks between stool and saliva samples of HC and RCC patients. HC: healthy controls, RCC: idiopathic chronic cough

| **Stool samples** | | | | | |
| --- | --- | --- | --- | --- | --- |
| **Rank** | **SumsOfSqs** | **MeanSqs** | **F.Model** | **R2** | **P value** |
| Phyla | 35.7551 | 35.7551 | 7.1696 | 0.2966 | 0.0024 |
| Classes | 41.0416 | 41.0416 | 3.5634 | 0.1732 | 0.0075 |
| Orders | 40.6009 | 40.6009 | 5.3606 | 0.2397 | 0.0047 |
| Families | 50.0684 | 50.0684 | 2.5049 | 0.1284 | 0.0081 |
| Genera | 65.8702 | 65.8702 | 1.9440 | 0.1026 | 0.0085 |
| **Saliva samples** | | | | | |
| **Rank** | **SumsOfSqs** | **MeanSqs** | **F.Model** | **R2** | **P value** |
| Phyla | 16.9949 | 16.9949 | 7.0451 | 0.2812 | 0.0003 |
| Classes | 36.7731 | 36.7731 | 7.2803 | 0.2879 | 0.0001 |
| Orders | 31.0901 | 31.0901 | 9.4603 | 0.3445 | 0.0002 |
| Families | 41.4992 | 41.4992 | 5.8162 | 0.2442 | 0.0001 |
| Genera | 49.2770 | 49.2770 | 5.5319 | 0.2350 | 0.0001 |

**Table S2.** Significant differentially abundant taxa in stool samples of RCC patients compared to HC. The table report the Log2FoldChange and adjusted p-values less than 0.05. HC: healthy controls, RCC: refractory chronic cough

| **Log2FoldChange** | **padj** | **Regulation** |  |  |
| --- | --- | --- | --- | --- |
| 1.5101 | 0.002 | *down* | Bacteroidota | **Phylum** |
| 3.1398 | 0.001 | *down* | Proteobacteria |  |
| 2.6831 | 0.018 | *down* | Gammaproteobacteria | **Class** |
| 3.3509 | 0.010 | *down* | Enterobacterales | **Order** |
| -3.2965 | 0.006 | *up* | Erysipelotrichaceae | **Family** |
| 2.1301 | 0.006 | *down* | Marinifilaceae |  |
| 6.0863 | 0.022 | *down* | Acidaminococcus | **Genus** |
| 24.6182 | 5.14e-15 | *down* | *Alloprevotella* |  |
| -2.0822 | 0.002 | *up* | *Anaerostipes* |  |
| -1.9160 | 0.002 | *up* | *Blautia* |  |
| 2.3611 | 0.034 | *down* | *Butyricimonas* |  |
| -24.2331 | 8.87e-15 | *up* | *CAG-352* |  |
| 4.7678 | 0.002 | *down* | *Clostridia_vadinBB60_group* |  |
| -2.7676 | 0.041 | *up* | *Enterorhabdus* |  |
| 25.5655 | 4.99e-16 | *down* | *Mitsuokella* |  |
| 2.6850 | 0.039 | *down* | *NA_ f Lachnospiraceae* |  |
| 2.3350 | 0.010 | *down* | *Odoribacter* |  |
| 2.3173 | 1.41e-05 | *down* | *Parabacteroides* |  |
| -2.1917 | 0.018 | *up* | *Streptococcus* |  |
| 6.8616 | 3.76e-07 | *down* | *Sutterella* |  |

**Table S3.** Significant differentially abundant taxa in saliva samples of RCC patients compared to HC. The table report the Log2FoldChange and adjusted p-values less than 0.05. HC: healthy controls, RCC: refractory chronic cough

| **Log2FoldChange** | **padj** | **Regulation** |  |  |
| --- | --- | --- | --- | --- |
| 1.0445 | 0.039 | *down* | Proteobacteria | **Phylum** |
| -1.4657 | 0.039 | *up* | Spirochaetota |  |
| 1.0732 | 2.81e-4 | *down* | Bacilli | **Class** |
| 1.2627 | 0.011 | *down* | Gammaproteobacteria |  |
| -1.3058 | 0.033 | *up* | Flavobacteriales | **Order** |
| 1.2927 | 0.013 | *down* | Micrococcales |  |
| 2.3505 | 0.007 | *down* | Burkholderiaceae | **Family** |
| 1.3573 | 0.007 | *down* | Micrococcaceae |  |
| 1.3404 | 0.007 | *down* | Pasteurellaceae |  |
| -1.5221 | 0.013 | *up* | Saccharimonadaceae |  |
| -2.2245 | 0.001 | *up* | Selenomonadaceae |  |
| 1.6549 | 0.010 | *down* | *Alloprevotella* | **Genus** |
| -1.4088 | 0.037 | *up* | *Capnocytophaga* |  |
| -1,8339 | 0.010 | *up* | *Dialister* |  |
| 2.2736 | 0.021 | *down* | *Lautropia* |  |
| -1.0797 | 0.037 | *up* | *Porphyromonas* |  |
| 1.3606 | 0.021 | *down* | *Rothia* |  |
| -3.3911 | 0.010 | *up* | Saccharimonadaceae |  |
| -2.0803 | 0.010 | *up* | Selenomonas |  |

**Table S4.** Fecal SCFAs abundances of HC and RCC patients. P-values were assessed the with Mann-Whitney test. SCFAs: short chain fatty acids, HC: healthy control, RCC: refractory chronic cough

| **Percentage of each fecal SCFA**  (median (IQR); %) | **CUD** | **HC** | **p value** |
| --- | --- | --- | --- |
| Acetic acid | 67.89 (5.91) | 67.09 (3.54) | 0.211 |
| Propionic acid | 14.29 (9.63) | 13.62 (3.69) | 0.968 |
| Butyric acid | 14.02 (2.32) | 11.16 (7.50) | 1.000 |
| isoButyric acid | 1.54 (1.88) | 1.38 (1.19) | 1.000 |
| isoValeric acid | 1.17 (1.61) | 1.04 (0.94) | 1.000 |
| 2-MethylButyric acid | 1.03 (1.78) | 1.01 (0.86) | 0.806 |
| Valeric acid | 1.84 (0.57) | 2.55 (1.06) | 0.414 |
